# Nucleus size and DNA accessibility are linked to the regulation of paraspeckle formation in cellular differentiation

**DOI:** 10.1186/s12915-020-00770-y

**Published:** 2020-04-22

**Authors:** Markus Grosch, Sebastian Ittermann, Ejona Rusha, Tobias Greisle, Chaido Ori, Dong-Jiunn Jeffery Truong, Adam C. O’Neill, Anna Pertek, Gil Gregor Westmeyer, Micha Drukker

**Affiliations:** 1 Institute of Stem Cell Research (ISF), Helmholtz Zentrum München, Neuherberg, Germany; 2 Institute of Stem Cell Research (ISF), iPSC Core Facility, Helmholtz Zentrum München, Neuherberg, Germany; 3 Comprehensive Pneumology Center (CPC), Helmholtz Zentrum München, Member of the German Center for Lung Research (DZL), Munich, Germany; 4 Institute of Biological and Medical Imaging (IBMI), Helmholtz Zentrum München, Neuherberg, Germany

## Abstract

**Background:**

Many long noncoding RNAs (lncRNAs) have been implicated in general and cell type-specific molecular regulation. Here, we asked what underlies the fundamental basis for the seemingly random appearance of nuclear lncRNA condensates in cells, and we sought compounds that can promote the disintegration of lncRNA condensates in vivo.

**Results:**

As a basis for comparing lncRNAs and cellular properties among different cell types, we screened lncRNAs in human pluripotent stem cells (hPSCs) that were differentiated to an atlas of cell lineages. We found that paraspeckles, which form by aggregation of the lncRNA *NEAT1*, are scaled by the size of the nucleus, and that small DNA-binding molecules promote the disintegration of paraspeckles and other lncRNA condensates. Furthermore, we found that paraspeckles regulate the differentiation of hPSCs.

**Conclusions:**

Positive correlation between the size of the nucleus and the number of paraspeckles exist in numerous types of human cells. The tethering and structure of paraspeckles, as well as other lncRNAs, to the genome can be disrupted by small molecules that intercalate in DNA. The structure-function relationship of lncRNAs that regulates stem cell differentiation is likely to be determined by the dynamics of nucleus size and binding site accessibility.

**Graphical abstract:**

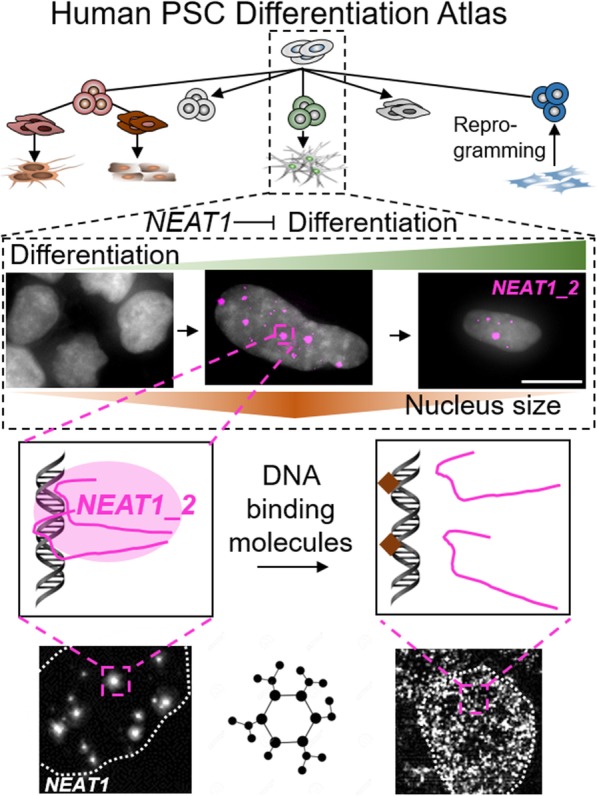

## Background

The human genome encodes for tens of thousands of lncRNAs [1], and we have just begun revealing their regulatory functions in development, disease, and homeostasis. Nuclear lncRNAs have been implicated in the regulation of gene expression in diverse ways, including the recruitment of chromatin-remodeling complexes of the SWI/SNF family [2], and interaction with Polycomb repressive complexes which modify histones [3]. Importantly, many nuclear lncRNAs have been reported to reside in condensates, named collectively “membraneless compartments” [4]. A notable example are paraspeckles which comprise the lncRNA *NEAT1* and RNA-binding proteins (RBPs) that influence gene expression by post-transcriptional regulation of splicing and polyadenylation [5, 6], as well as by interaction with the SWI/SNF complex that remodels nucleosomes [7]. Similarly, the lncRNA *MALAT1*, which also forms condensates, has been shown to regulate gene expression by interactions with splicing factors [8]. Despite advancements in understanding the composition and formation of lncRNA-protein condensates [9, 10] and separately the functions of RBPs, for example, TDP-43 in the regulation of alternative polyadenylation [11], the regulation mediated together by the aggregation of lncRNAs, RBPs, and other factors is still not well understood.

Nuclear lncRNAs, and presumably lncRNA condensates such as paraspeckles, can be tethered to double-stranded (ds) DNA by forming RNA-dsDNA triple helix complexes, and such interactions have been proposed to rely on sequence-specific base-pairing interactions [12, 13]. Whether such interactions are the basis for the locations of hundreds of associations with open chromatin regions, which were reported for lncRNAs such as *NEAT1* and *MALAT1,* is an open question [14]. A strategy that could assist in elucidating the underlying mechanisms of interactions between lncRNAs and chromatin is the identification of compounds that alter the structure of dsDNA. Plausible types of small molecules in this regard include DNA-binding compounds from the Hoechst family [15], and a host of other minor groove-associating molecules that are used for chemotherapy, such as actinomycin D (ActD) [16].

The use of hPSCs for studying the functions of lncRNA condensates is advantageous in several respects: first, the differentiation of hPSCs is accompanied by changes of genome architecture [17] that create opportunities to study the formation of lncRNA condensates in cell fate transitions, as we have shown recently for paraspeckles [18]. Second, differentiation protocols allow the generation of cell types from the three embryonic germ layers in order to analyze and compare general and cell type-specific regulation, for example, using differentiated neurons, hepatocytes, and cardiomyocytes. In this respect, we have recently observed that the number of paraspeckles exhibited by individual cells during early differentiation of mouse and human PSCs is variable despite the robustness of differentiation protocols in creating homogenous preparations of differentiated cells [18], and similar observations have been made previously in tumor cell lines [19]. This indicates that an unknown form of structural regulation causes the seemingly random appearance of paraspeckles in cells, which could be linked to general mechanisms that regulate the vastly different number of paraspeckles observed in different cell types and upon exit from pluripotency [18, 20, 21].

Our objective here was to expand the understanding of the fundamental basis for formation of lncRNA-protein condensates and their functions. We first screened lncRNAs that were previously shown to concentrate in foci and used hPSC differentiation protocols of the three germ layers in order to characterize lncRNAs that exhibit dynamic expression patterns. This led us to analyze in detail the formation of *NEAT1* condensates, namely paraspeckles, and to test whether small molecules can disintegrate them in vivo. In order to identify the intrinsic regulation of paraspeckles, we differentiated an atlas of cell types, and then applied dsDNA minor groove-associated small molecules. By doing so, we dissected the kinetics of paraspeckle formation in cellular differentiation and discovered that the amount of paraspeckles is scaled in relation to cells’ nucleus size. We further validated the scaling principle using different human somatic cells, fibroblasts that underwent reprogramming, and murine cells. Moreover, we identified the first small molecules that disintegrate paraspeckles and other lncRNA condensates, presumably by perturbing RNA-dsDNA triple helix structures. Beyond the importance of these molecules for studying lncRNA condensates and their tethering to the chromatin, some of these small molecules are approved drugs for chemotherapy, which raises the possibility that they could be repurposed for treating diseases involving aberrant formation of lncRNA condensates. Finally, we demonstrated that paraspeckles are involved in the differentiation of hPSCs by slowing down the process. Thereby, we connected paraspeckles to nuclear scaling, DNA tethering, and developmental regulation and, more broadly, identified reagents for lncRNA condensate research and medicine.

## Results

### Dynamics of nuclear lncRNAs in the differentiation of human PSCs

**Fig. 1:**
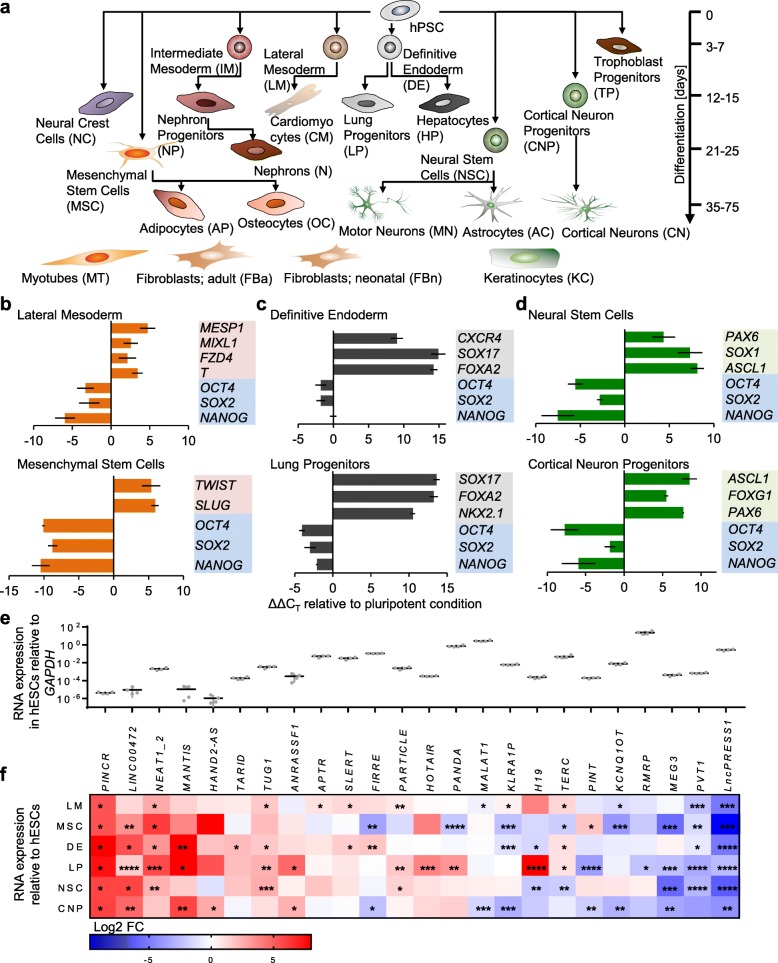
Characterization of developmentally regulated lncRNAs.** a** A scheme illustrating the cell types that were produced in this study by differentiation of hPSCs. Starting with undifferentiated cells at the top, hPSCs were differentiated to precursors of the germ layers, embryonic and extraembryonic progenitors, and terminally differentiated cells. The lineage and approximate developmental distance was estimated based on the expression of developmental markers as outlined below and in Additional file 1: Figure S1. In addition, primary preparations of keratinocytes, fibroblasts (adult and neonatal origin), and myotubes were analyzed. **b–d** RT-qPCR analysis of selected lineage markers corresponding to lateral mesoderm, and mesenchymal stem cells (**b**); definitive endoderm and lung progenitors (**c**); and neural progenitors and cortical neuron progenitors (**d**). Pluripotency genes *OCT4*, *SOX2*, and *NANOG* were analyzed in all samples. *n* = 2 independent experiments. **e, f** The absolute (**e**) and relative (**f**) expression of nuclear lncRNAs in undifferentiated human ESCs and germ layer and tissue progenitors as in **b–d** based on RT-qPCR analysis. *n* = 2 (CNP), 3 (LM, MSC), 4 (LP, pluripotent, NSC), 5 (DE) independent experiments, error bars represent standard deviation, *p* values were calculated by unpaired *t*-test; * p < 0.05, ** *p* < 0.01, *** p < 0.001, **** p < 0.0001. Cells at different passages were used for replicates. Abbreviations: LM: lateral mesoderm, MSC: mesenchymal stem cells, DE: definitive endoderm, LP: lung progenitors, CNP: cortical neuron progenitors, NSC: neural stem cells

To conduct an unbiased assessment of the association of cell types and developmental stages with the expression and condensation of nuclear lncRNAs, we differentiated human PSCs to numerous lineages (Fig. 1a). We first optimized the differentiation protocols of lateral mesoderm and mesenchymal stem cells (MSCs), definitive endoderm and lung progenitor cells, and neural stem cells (NSCs) and cortical neuron progenitors, which represented, respectively, early and late stages of differentiation of the three germ layers mesoderm, endoderm, and ectoderm. We observed the upregulation of lateral mesoderm markers *MESP1*, *T* (*Brachyury*), *FZD4*, and *MIXL1*, and transcription factors *TWIST* and *SLUG* which regulate the epithelial-to-mesenchymal transition of MSCs, as well as surface markers that are characteristic for MSCs (Fig. 1b and Additional file 1: Figure S1a). When differentiated to definitive endoderm, we detected the upregulation of *SOX17*, *FOXA2*, surface markers *CXCR4*, *CD117*, and *EPCAM*, and later of the master lung transcription factor *NKX2.1* (Fig. 1c and Additional file 1: Figure S1b-d). Moreover, the upregulation of *PAX6*, *SOX1*, *ASCL1*, *NESTIN*, and *FOXG1* mRNAs and respective proteins confirmed the differentiation to NSCs and cortical neuron progenitors, respectively (Fig. 1d and Additional file 1: Figure S1e, f). Finally, in all cell types, we observed the downregulation of the pluripotency factors *OCT4*, *SOX2*, and *NANOG* (Fig. 1b–d), which confirmed their differentiation.

We screened 27 lncRNAs (Additional file 5: Table S3) that participate in the regulation of gene expression [22], including some that are known to form condensates [23], and found that the vast majority of them, 24, were either up- or downregulated upon differentiation, but mostly not in an obvious differentiation stage-specific manner (Fig. 1e, f). A striking example of lineage-specific regulation was the induction of *H19* (*p* < 0.0001) in lung progenitor cells. Importantly, several lncRNAs were upregulated in all germ layers and stages including *PINCR*, *LINC00472*, and *NEAT1_2* (*p* < 0.05 in ≥ 5 lineages), in contrast to lncRNAs such as *MALAT1* which were mostly constitutively expressed. We chose *NEAT1_2* for an in-depth analysis of condensation behavior because it is well known to form paraspeckles that have been linked to the regulation of development and differentiation [18, 21].

### Atlas of paraspeckle trajectories during cell fate transitions 

As a basis for identifying cellular features that are associated with the formation of paraspeckles, we differentiated hPSCs to more than 20 cell types and quantified foci of *NEAT1_2*, the facultative marker of paraspeckles [19]*,* using single-molecule FISH (smFISH). Mesoderm was represented by differentiating MSCs to adipocytes and osteocytes, lateral mesoderm to cardiomyocytes, and intermediate mesoderm to nephron progenitors and matured nephrons; definitive endoderm cells were differentiated to hepatocytes and lung progenitors; NSCs, which belong to ectoderm, were differentiated to motor neurons and astrocytes, and cortical neuron progenitors were cultured to a mature state; neural crest progenitors, which give rise to multiple lineages that migrate throughout the body [24], were produced from neurospheres [25]. Extraembryonic tissues were represented by trophoblast progenitors differentiated from hPSCs [26], and myotubes, keratinocytes, and fibroblasts were derived from primary tissues (Fig. 1a).

**Fig. 2:**
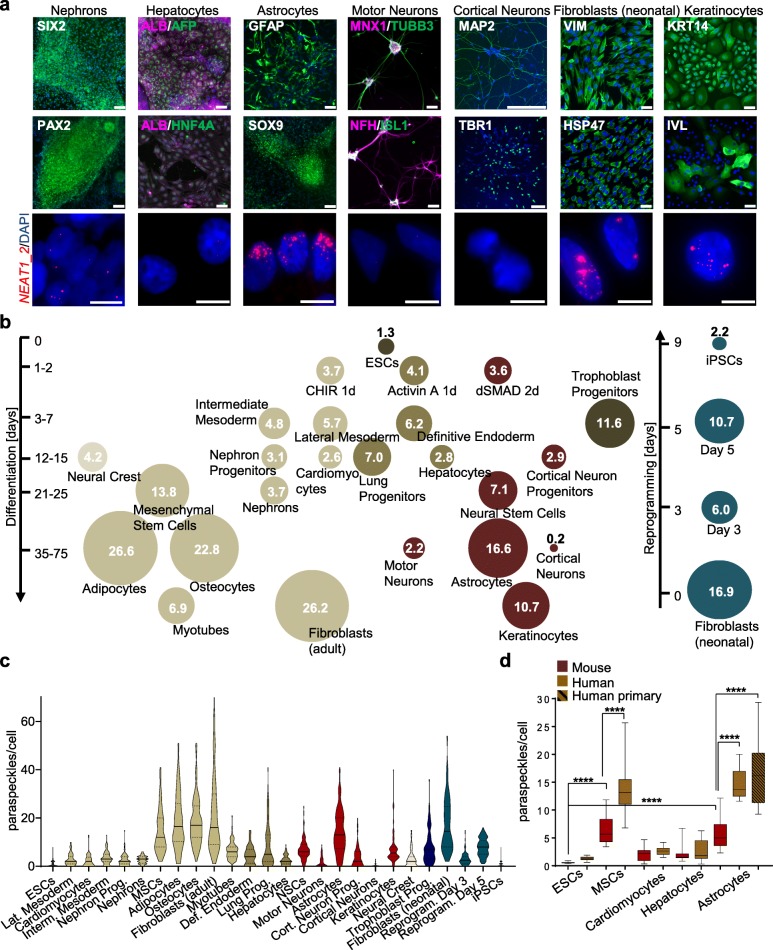
Characterization of paraspeckles in a panel of cell types and differentiated states. **a** Representative immunocytochemistry images of terminally differentiated cell preparations stained with antibodies, specific for markers of the respective cell types (scale bar upper panels: 50 μm) and analyzed by smFISH with *NEAT1_2* probe (bottom panel, probe in red, DAPI staining in blue; scale bar: 10 μm). **b** A summary of the number of paraspeckles in diverse developmental and terminally differentiated cell types, and during reprogramming of human neonatal fibroblasts (Additional file 2: Figure S2i-k). Size of circles corresponds to the average number of paraspeckles in the different cell types quantified by automated spot (foci) detection in a total of 200–2000 cells per type representing three independent experiments (single data points and statistics in Additional file 2: Figure S2c) **c** Violin plots depicting the number of paraspeckles in 100 single cells from all tested human cell types based on **b**, black line represents mean value and dashed lines represent the quartiles. **d** Quantification of paraspeckle in primary murine cell types (*n* = 3 independent replicates using ESCs, or three tissue preparations for the other cell types with 10–28 images in total per condition). Representative images in Additional file 2: Figure S2f shown next to the corresponding human cell populations from **b**. Error bars represent standard deviation, **** *p* < 0.0001 unpaired *t*-test

We based cell type classifications on the analysis of characteristic proteins, transcripts, and additional features as follows: differentiated MSCs exhibited lipid droplets and calcium deposits, which are associated with adipocytes and osteocytes, respectively (Additional file 1: Figure S1g, h); differentiation of lateral mesoderm progenitors led to upregulation of cardiomyocyte progenitor markers including *NKX2.5* and *ISL1* (and spontaneous beating was observed) and downregulation of early mesoderm markers *T* and *MESP1* (Additional file 1: Figure S1i); markers of the developing kidney, *SIX2*, *PAX2 CDH5*, *WT1*, and additional nephron progenitor markers were expressed (Fig. 2a and Additional file 1: Figure S1j, k). Endoderm differentiation was apparent by expression of liver markers *AFP*, *ALB*, and *HNF4A* (Fig. 2a and Additional file 1: Figure S1l). Characterization of the neuronal cell populations was based on the formation of TUBB3 and NFH-positive axons in the case of motor neurons, MAP2-positive axons in the case of cortical neurons, and GFAP-positive star-like projections in the case of astrocytes (Fig. 2a). Moreover, these cell populations expressed the characteristic transcription factors *MNX1*, *ISL1*, *TBR1*, and *SOX9*, respectively (Fig. 2a), which were confirmed by analysis of the mRNAs together with neuronal markers *CHAT* and *TBR2* as well as markers of astrocytes *SLC1A2* and *SLC1A3* (Additional file 1: Figure S1m-o). Finally, the identity of fibroblasts and keratinocytes was validated by expression of *VIM* / *HSP47* and *KRT14* / *IVL*, respectively (Fig. 2a).

Inspection of the cell atlas confirmed the previous observations that the number of paraspeckles increases when hPSCs exit the pluripotent state [18, 27]. Moreover, it was apparent that the increase in the number of paraspeckles during exit from pluripotency is a general phenomenon that encompassed progenitors of the three germ layers, neural crest progenitors, and extraembryonic trophoblast progenitors (Fig. 2b and Additional file 2: Figure S2a, c). Because these types of progenitors exhibited vastly different transcriptional programs and epigenetic landscapes [28, 29], these results indicated that the regulation of paraspeckle formation is connected to a general mechanism of nuclear organization. Formation of heterochromatin upon differentiation is one such mechanism [30], however, the fact that the number of paraspeckles varied considerably between related types of cells spoke against it. For instance, differentiated cell types that belong to the mesoderm lineage displayed either 2.6–5.7 paraspeckles per cell on average, or 13.8–26 in MSCs and their adipocyte and osteocyte progeny (Fig. 2b and Additional file 2: Figure S2a-c). Similarly, in the neural lineages, there was a striking difference between the number of paraspeckles in the neurons and astrocytes that were derived from NSCs (Fig. 2b, c and Additional file 2: Figure S2c). Moreover, hepatocytes and lung progenitors exhibited differences in the number of paraspeckles despite their close endoderm origin. Additional evidence indicating that the regulation of the number of paraspeckles is not overtly determined by cell lineages or timing of differentiation were (a) the weak correlation between the number of paraspeckles and the time point of differentiation (Additional file 2: Figure S2e), (b) the oscillations in the number of paraspeckles during cellular reprogramming (the reverse process of differentiation) (Fig. 2b and Additional file 2: Figure S2i-k), and (c) that adult dermal fibroblasts exhibited significantly more paraspeckles compared to neonatal foreskin fibroblasts (Fig. 2b and Additional file 2: Figure S2c). Evidence indicating that the number of paraspeckles represents cell-intrinsic regulation included passaging of neural stem cells which did not change the number of paraspeckles (Additional file 2: Figure S2d) and that primary tissue-derived human astrocytes exhibited similar amount of paraspeckles as the in vitro differentiated astrocytes (Fig. 2d).

To further analyze cellular traits that influence paraspeckle formation, we asked whether similar amounts of paraspeckles appear in equivalent types of cells from the mouse. Strikingly, we found that despite the trends being similar, i.e., astrocytes exhibiting greater amounts of paraspeckles compared to cardiomyocytes, hepatocytes, and ESCs, the number of paraspeckles in the respective types of cells in the mouse was significantly lower (Fig. 2d and Additional file 2: Figure S2f). We substantiated these results by showing that the general correlation between the signal intensity of smFISH and the number of paraspeckles counted in human and mouse cells was very high and that the level of *NEAT1_2* transcript was generally correlated (Additional file 2: Figure S2g, h). Altogether, we concluded that cellular differentiation creates diverse patterns of paraspeckle kinetics which are not overtly correlated with developmental lineages or timing.

### Paraspeckle amount correlates with the size of the nucleus

**Fig. 3:**
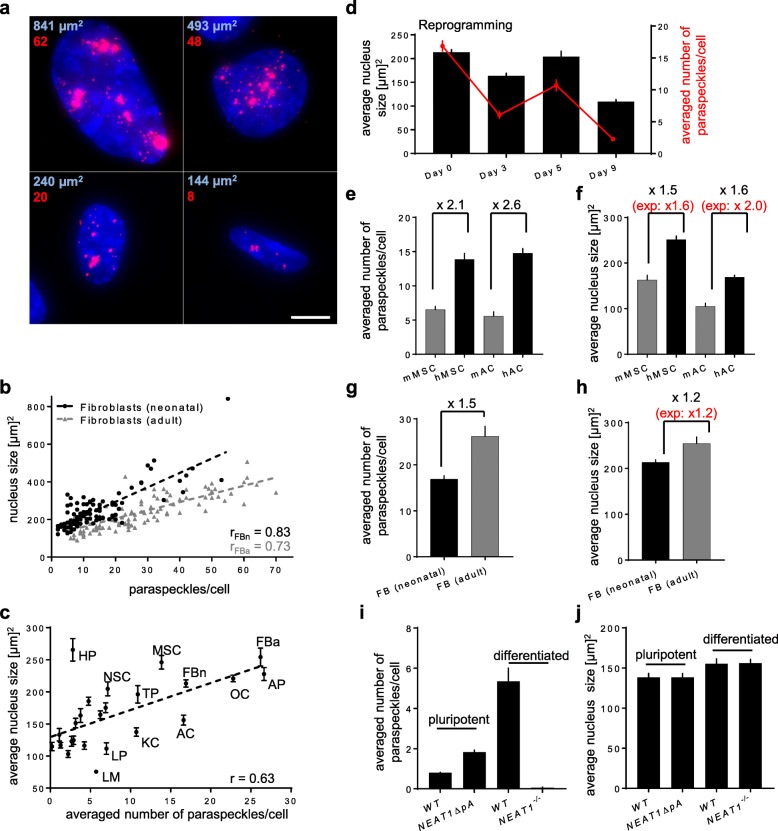
Paraspeckle formation correlates with the size of nucleus.** a** Images and quantification of nuclear area (μm^2^) by DAPI staining (blue) and number of *NEAT1_2* foci analyzed by smFISH (red) of representative human adult neonatal fibroblasts that exhibited different sizes (scale bar: 10 μm). **b** Analysis of the correlation between the number of paraspeckles and nucleus size of 100 human neonatal (black) and adult (gray) fibroblasts. **c** Analysis of the correlation between the averaged number of paraspeckles and average nucleus size per cell in 24 cell types analyzed in the atlas database represented in Fig. 2b. **d** Average nucleus size (black) and number of paraspeckles (red; based on Fig. 2b) analyzed during reprogramming of human neonatal fibroblasts. **e–h** Averaged number of paraspeckles per cell (**e**, **g**) based on Fig. 2b, d and average nuclear size (**f**, **h**) in mouse (gray) and human (black) MSCs and astrocytes (AC), as well as in adult (gray) and neonatal (black) fibroblasts. Numbers on top are the fold changes between the respective cell types from human and mouse. The numbers in red represent predicted fold changes based on slope of regression line in **c**. **i**, **j** Average number of paraspeckles per cell (**i**) and average nucleus size (**j**) of *NEAT1*^*−/−*^, *NEAT1ΔpA* and *WT* hESCs in pluripotent condition or differentiated by addition of retinoic acid for 3 days to induce paraspeckle formation [18]. The generation of *NEAT1*^*−/−*^ hESCs is outlined in Fig. 5a–d. Nucleus size represents the averaged value of 7–14 images per cell type from two independent experiments with 10–100 cells per image (details in methods). Error bars represent standard error of the mean. r in **b**, **c** represents Pearson’s correlation coefficient and dashed line is the linear regression line

One characteristic of the nucleus that varies drastically between different cell types is its size [31]. We therefore used our cell atlas image database to ask whether size scaling of the nucleus can explain the different amounts of paraspeckles in different cell types. Strikingly, we noticed a positive correlation between the number of paraspeckles and the size of nuclei when inspecting individual fibroblasts (Fig. 3a, b). Moreover, we noted a greater amount of paraspeckles in fibroblasts that were derived from adult compared with neonatal foreskin fibroblasts (Fig. 3b and Additional file 2: Figure S2c), which could be the result of an increase in cellular senescence accompanied by nucleus size increase [32]. These results prompted us to investigate whether the size of the nucleus is in general predictive for paraspeckle quantity in different types of cells (Fig. 2). Strikingly, analyzing the nucleus size in all the cell types of the atlas revealed a correlation with the number of paraspeckles (Fig. 3c). Furthermore, we found that the oscillating pattern of paraspeckle formation during reprogramming could be explained by changes in the average size of the nucleus during the process (Fig. 3d). This led us to hypothesize that the differences in paraspeckle amount between human and mouse astrocytes and MSCs (Fig. 2d) are due to nucleus size differences. Indeed, adjusting the number of paraspeckles to nucleus size differences between human and mouse MSCs and astrocytes showed that the normalized values of paraspeckles are similar between the species (Fig. 3e, f). Finally, the differences in paraspeckle numbers between human neonatal and adult fibroblasts could be explained in the same way by nucleus size changes (Fig. 3g, h). These results provided a first explanation for the high degree of variability in the number of paraspeckles observed between cells of the same type, between different types of cells and between species.

Finally, to assess whether the size of the nucleus determines the amount of paraspeckles or vice versa, we analyzed *NEAT1*^*−/−*^ (introduced in detail in Fig. 5a) and *NEAT1ΔpA* hESCs which either lacked paraspeckles or exhibited a twofold increase in the amount of paraspeckles due to the deletion of the internal polyA site [18] (Fig. 3i). Analyzing the size of nuclei did not reveal differences between *NEAT1*-modified cell lines compared to wildtype (Fig. 3j), thus we concluded that it is the nucleus size that likely determines the amount of paraspeckles.

### DNA accessibility is required for paraspeckle assembly

**Fig. 4:**
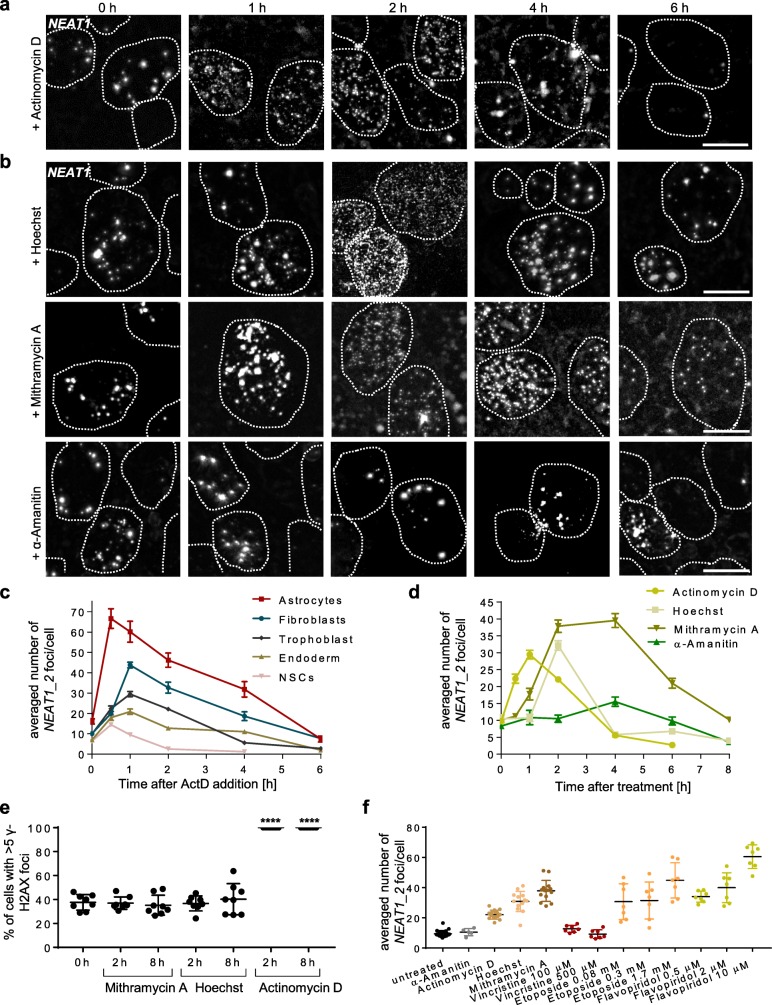
Treatment with DNA-binding small molecule compounds promotes paraspeckle disassembly. **a**, **b** Representative images of *NEAT1_2* smFISH after treatment of cells by 2 μM ActD (**a**), 100 μg/ml Hoechst 33342, 5 μM mithramycin A, and 50 μM α-amanitin (**b**) in trophoblast progenitors produced by 3-day BMP4 treatment of hESCs. Dashed lines show the locations of the borders of the nuclei. Scale bar: 10 μm. **c** Analysis of the averaged amount of *NEAT1_2* foci following ActD treatment in five different cell types. Images in Additional file 3: Figure S3a. **d** Analysis of the averaged amount of *NEAT1_2* foci in trophoblast progenitors following treatment by the four compounds shown in **a**, **b**. **e** Quantification of γ-H2AX foci (associated with DNA double-strand breaks) in trophoblast progenitors and after addition of DNA-binding compounds. Representative images in Additional file 3: Figure S3c. **f** Analysis of the averaged amount of *NEAT1_2* foci in trophoblast progenitors following 2 h of treatment by the compounds as in **a, b** and different concentrations of the chemotherapeutic reagents vincristine, etoposide, and flavopiridol. DNA-binding and transcriptional inhibition properties of the compounds are listed in Additional file 3: Figure S3d. Error bars in **c**, **d** represent standard error of the mean and standard deviation in **e**, **f**. Seven images were analyzed in **e**, **f** and 14 in **c**, **d**, representing two independent replicates using cells of different passages. **** *p* < 0.0001 unpaired *t*-test

The broad range of the number of paraspeckles in cells with different nucleus size led us to interrogate what common traits could regulate their structural similarity across different types of cells. Because several lncRNAs, including *NEAT1_2* and *MALAT1,* have been associated with formation of RNA-dsDNA triple helix structures through base pairing in the major groove [13, 33], we hypothesized that conformational changes of the DNA helix could perturb paraspeckles. We therefore tested whether small molecules such as ActD that binds the minor groove in dsDNA can promote the disassembly of paraspeckles. Strikingly, we noted the appearance of numerous small *NEAT1_2* speckles that peaked between 1 and 2 h in diverse types of cells after treatment by ActD, including trophoblast progenitors, neural stem cells, and definitive endoderm progenitors that were derived from hPSCs, as well as in primary astrocytes and adult dermal fibroblasts (Fig. 4a and Additional file 3: Figure S3a). Importantly, the numbers of small *NEAT1_2* speckles matched the number of paraspeckles observed in the respective cell types (Figs. 2b and 4c), indicating that ActD induced the disintegration of paraspeckles. Contrarily, we noted that core paraspeckle proteins, namely SFPQ and NONO localized to perinucleolar caps after addition of ActD (Additional file 3: Figure S3b) which is in line with previous observations that reported perinucleolar localization of paraspeckle proteins after transcriptional inhibition [34] and during cell division [35] when *NEAT1_2* is downregulated. Collectively, these results indicated that the disintegrated speckles of *NEAT1_2* that were produced by ActD treatment were not functional.

Based on these findings, we further tested the effects of Hoechst 33342 and mithramycin A which induce conformational changes of the dsDNA helix by binding to the minor groove [36, 37]. These compounds led to the appearance of small *NEAT1_2* speckles that exhibited similar patterns of accumulation and decay as by ActD (Fig. 4b, d). Importantly, α-amanitin (selective inhibitor of RNA polymerases II, III, and IV) did not induce immediate paraspeckle disintegration (Fig. 4b, d), which ruled out the possibility that inhibition of RNA polymerases was the underlying cause. Nevertheless, the disappearance of *NEAT1_2* following several hours of continuous treatment was likely due to other mechanisms that interfere with transcription as it has been shown that inhibition of transcription impairs paraspeckle formation [38, 39]. It is known that some small DNA-binding molecules can induce double-strand breaks [40], which can be analyzed by the appearance of γ-H2AX foci [41]. We found this to be the case following ActD treatment but not following Hoechst or mithramycin A (Fig. 4e and Additional file 3: Figure S3c). We concluded that immediate paraspeckle disintegration is not mediated by DNA damage or inhibition of RNA polymerases.

Because ActD and mithramycin A are used in chemotherapy protocols to treat several types of cancer [42], we were interested whether paraspeckle disintegration could be induced by other chemotherapeutic reagents. We tested this by treating the cells with the microtubule inhibitor vincristine [43], the topoisomerase II inhibitor etoposide [44], and flavopiridol, an inhibitor of cyclin-dependent kinases [45]. We observed a significant increase in small *NEAT1_2* speckles after treatment by etoposide and flavopiridol, but not by vincristine (Fig. 4f), and since only the first two were shown to bind dsDNA [46, 47], this supported our conclusion that DNA binding by small molecules induced paraspeckle disintegration (Additional file 3: Figure S3d).

Finally, to test whether small DNA-binding molecules can in general disintegrate lncRNA condensates, we analyzed *MALAT1* speckles by smFISH after ActD treatment. Strikingly, we found that *MALAT1* speckles disintegrated with similar kinetics as paraspeckles (Additional file 3: Figure S3e, f). We concluded that dsDNA helix binding serves as structural basis for assembly and maintenance of paraspeckles and other nuclear lncRNA condensates.

### *NEAT1_2* regulates differentiation

**Fig. 5:**
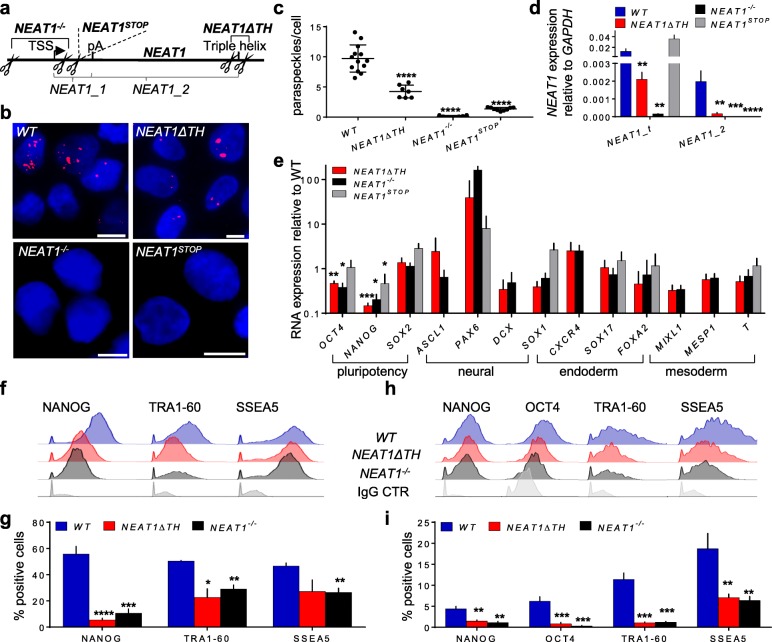
*NEAT1_2* depletion increases differentiation potential. **a** The strategy of generating *NEAT1*^*−/−*^, *NEAT1*^*STOP*^, and *NEAT1ΔTH* hESC clones by CRISPR/Cas9. **b–d** Representative images (**b**) and quantification of paraspeckles by smFISH with *NEAT1_2* (red) probe (**c**) or RT-qPCR (**d**) in parental (WT), *NEAT1ΔTH*, *NEAT1*^*−/−*^, and *NEAT1*^*STOP*^ hESCs after 3 days of differentiation induced by retinoic acid (**b**, **c**) or spontaneous differentiation (**d**). Primers for total *NEAT1* targeted both *NEAT1* isoforms. DAPI staining in blue; scale bar: 10 μm. Low levels of paraspeckles were assigned to *NEAT1*^*−/−*^ and *NEAT1*^*STOP*^ hESCs due to background signals. **e–i** RT-qPCR of pluripotency and differentiation markers (**e**), and flow cytometry of pluripotency markers after WT, *NEAT1ΔTH* and *NEAT1*^*−/−*^ hESCs were spontaneously differentiated for 3 days (**f**, **g**) or after 4 days of neuroectoderm differentiation (**h**, **i**). *NEAT1*^*STOP*^ hESCs were included in **e**. *n* (# of experiments / # of clones) = 2/2 in **d**, 2/3 in **e**, **g** (except *NEAT1*^*STOP*^ which is 3/2) and 1/3 in **i**. Error bars represent standard deviation in **c**–**e** or standard error of the mean in **g**, **h**. * p < 0.05, ** p < 0.01, *** p < 0.001, **** p < 0.0001, unpaired *t*-test

Our results revealed that paraspeckles are dynamically regulated in cellular differentiation by the size of the nucleus and DNA accessibility. In order to analyze their functional role during the differentiation of germ layer progenitors, which commonly upregulate paraspeckles upon exit from pluripotency (Fig. 2), we created a series of genetically edited lines. This included deletion of the promoter, the transcription start site, and downstream sequences of *NEAT1* in one line, deletion of the triple helix (TH) sequence that resides in the 3’ end of the gene, and insertion of poly (A) stop signal downstream to the transription start site. Because the TH is required for processing of *NEAT1_2* [39], we hypothesized that its deletion will create a knock-down phenotype. Accordingly, we did not observe any paraspeckles and expression of *NEAT1_2* in the *NEAT1*^*−/−*^ line, and 57% less paraspeckles in the *NEAT1ΔTH* line after spontaneous differentiation (Fig. 5a–d).

By analyzing the expression of developmental markers, we found that *NEAT1*^*−/−*^ and *NEAT1ΔTH* clones exhibited normal characteristics of undifferentiated cells with the exception of premature upregulation of *FOXA2* and *PAX6* (Additional file 4: Figure S4a). Remarkably, the induction of spontaneous differentiation revealed acceleration of the downregulation of pluripotency transcription factors *OCT4* and *NANOG* and cell surface markers *TRA1–60* and *SSEA5* in *NEAT1*^*−/−*^ and *NEAT1ΔTH* cells compared to the parental cells one day following the appearance of paraspeckles (Fig. 5e–g and Additional file 4: Figure S4b, c). A similar acceleration was observed during neuroectoderm differentiation, albeit the gene expression patterns became similar to the parental line at later stages of differentiation (Fig. 5h, i and Additional file 4: Figure S4d). As we observed similar phenotypes for *NEAT1*^*−/−*^ and *NEAT1ΔTH* cell lines (Fig. 5e–i and Additional file 4: Figure S4a-d), despite the latter being able to produce the short isoform (Fig. 5d), we hypothesized that *NEAT1_1* is dispensable for differentiation of germ layer progenitors. Indeed, analyzing the differentiation of a line that harbored a deletion of the internal polyadenylation site in *NEAT1* (*NEAT1ΔpA*), and hence was not capable of expressing *NEAT1_1* [18], did not reveal a difference in the up- and downregulation of differentiation and pluripotency genes (Additional file 4: Figure S4e-g). Furthermore, *NEAT1*^*STOP*^ cells exhibited accelerated downregulation of the pluripotency transcription factors *OCT4/NANOG* and the surface markers *SSEA4/SSEA5/TRA1-60* during spontaneous differentiation (Additional file 4: Figure S4h-k), similar to the *NEAT1*^*−/−*^ and *NEAT1ΔTH* lines.

We concluded that only condensated *NEAT1*, namely paraspeckles, are functionally important during spontaneous or neural differentiation of hPSCs by slowing down the process, but cells can compensate for the loss of paraspeckles by further differentiation.

## Discussion

LncRNAs are predominantly localized in the nucleus [48] where some have been implicated in the architectural organization of chromatin by formation of condensates [22]. In this regard, *NEAT1* is paradigmatic for studying lncRNA condensation because it is the vital scaffold for the formation of paraspeckles together with RBPs and chromatin factors that have been well-characterized [6, 9, 49]. Despite major advancements in understanding the molecular structure of paraspeckles and the regulation of *NEAT1* RNA [39, 50], basic questions pertaining to the regulation of paraspeckle formation, quantities in cells, and their molecular and cellular functions remain open. Here, we made several observations that shine light on these questions.

First, we show that the amount of paraspeckles in cells within a population, or among different cell populations, is generally proportionally scaled to the size of the nucleus. This finding could have important implications, for example, it could indicate that paraspeckles are engaged in feedback(s) that slow-down cellular processes when nuclei reach set size(s), such as energy production. In this regard, paraspeckles have been implicated recently in the regulation of mitochondria homeostasis [51], and their upregulation following viral infection [9] and in tumor cells [52] could be further indications of this connection [53]. Interestingly, we noted that certain cell types deviate substantially from the nucleus size rule, most notably hepatocytes, which could indicate complex forms of regulation by paraspeckles in specific types of cells. The nucleus sizing effect could also indicate that the regulation of paraspeckles is more pronounced in human stem cell differentiation, and plausibly also in human development, compared to the equivalent processes in the mouse since nuclei in mouse cells are often significantly smaller and contain fewer paraspeckles compared to corresponding human cell types. In line with this idea is the fact that paraspeckles are not vital for mouse development past the blastocyst stage [20, 21], although their knock-down in embryos at the gastrulation stage increases the odds of developmental perturbations [18]. Collectively, the new connection that we found between the scaling of the nucleus to paraspeckle-mediated regulation emphasizes that dissecting the molecular and cellular functions of paraspeckles should take into account processes that could be affected by nucleus size scaling. It is important to note that our data revealed only a correlative link between the size of the nucleus and the amount of paraspeckles, and further experiments should elaborate the manipulation of nucleus size in relation to the effect on paraspeckle formation in order to establish a causal relationship.

Second, by applying small molecules that can associate with the minor groove of dsDNA, we found strong evidence that paraspeckles and other nuclear speckles are tethered to the DNA, most likely by forming triple helix structures [13, 33]. The fact that molecules with vastly different structures can promote paraspeckle disintegration indicates that conformational changes of DNA are the common cause. These findings imply that numerous lncRNAs are anchored in the genome to regulate its biochemical functions. If this is proven by future studies, it would indicate that aberrant regulation of tethered lncRNA condensates could be involved in human disease. In line with this prediction, a growing list of lncRNAs has been associated with different disorders, including diabetes [54] and cancer [55]. Therefore, our finding of small molecules that disintegrate tethered lncRNA condensates could lead to development of potential novel therapeutic approaches that are based on lncRNA disintegration. The fact that several of the molecules that we implicated here in the disintegration of paraspeckles and other nuclear speckles are approved chemotherapies, suggests that their clinical assessments could be expedited. On the other hand, as we implicated common chemotherapies in disintegration of lncRNA foci, it is important to consider the possibility that on-target and/or side effects in cancer therapy are mediated by changes of the structures of lncRNAs. Therefore, our results could be important for creating a new path to understand the mode-of-action in the treatment of certain cancers, for example, by actinomycin D, etoposide, and mithramycin A, which are commonly used in the treatment of osteosarcoma [42, 56]. Taken together with our assessment of the functional importance of paraspeckles in stem cell regulation, we suggest that their involvement in slowing down differentiation processes is connected to dsDNA tethering.

## Conclusion

We provide a comprehensive quantification of paraspeckles in an atlas of human cell types. Based on this, we deduce common cellular features that indicate how paraspeckle formation is regulated, namely by cells’ nucleus size which positively correlates with the number of paraspeckles and DNA tethering. Perturbing the latter by small dsDNA-binding molecules disintegrates paraspeckles and other chromatin-embedded lncRNAs. Lastly, we show that paraspeckles are important for early stem cell differentiation, and thus their function in this intricate regulation may be connected to the increase of the nucleus size, which takes place during the differentiation of PSCs, as well as to architectural changes of chromatin that could affect their tethering.

## Methods

### PSC culture

Human ESCs of the H9 line (WiCELL Research Institute) and iPSCs were cultured in StemMACS iPS-Brew XF (Miltenyi Biotec) and passaged by StemMACS Passaging Solution (Miltenyi Biotec) on tissue culture-treated plates (Sigma) coated with Matrigel (Thermo Fisher Scientific) diluted 1:100 in DMEM/F-12 (Thermo Fisher Scientific). All differentiation experiments were carried out with H9 cells, except lung progenitor and cortical neuron differentiation, which were performed with iPSC lines, namely NKX2.1-P2A-eGFP [57] and foreskin fibroblast-derived iPSCs [58], respectively. For paraspeckle measurements in trophoblast progenitors and neural crest cells, we used differentiation protocols, as previously described [26, 59].

### Fibroblast reprogramming

The reprogramming of human neonatal dermal fibroblasts was performed using StemRNA 3rd Gen Reprogramming Kit (Reprocell) according to the manufacturer’s protocol. The RNA transfection cocktail included synthetic, non-modified RNA of reprogramming factors OCT4, SOX2, KLF4, cMYC, NANOG, and LIN28A, immune evasion mRNAs of E3, K3, B18, and reprogramming-enhancing, mature, double-stranded microRNAs from the 302/367 cluster. 1.0 × 10^4^ fibroblasts were plated per 60 mm organ culture dish (Corning), and reprogramming was started the following day by lipofection of the mRNA cocktail and incubation overnight. Transfections were repeated daily for 3 days and at day 9, distinct iPSC colonies were forming.

### Spontaneous differentiation

One day prior to the beginning of spontaneous differentiation, 5.0 × 10^5^ cells that were dissociated using Accutase (Sigma) were transferred to one Matrigel-coated well of a 12-well plate with StemMACS iPS-Brew XF and 10 μM Y-27632 (R&D Systems). After 24 h, the medium was replaced with medium containing 20% KnockOut Serum Replacement (KSR), 1% GlutaMAX, 1% non-essential amino acids (NEAA), and 0.1 mM beta-mercaptoethanol (all Thermo Fisher Scientific). Fresh medium was applied daily for up to 3 days.

### MSC, adipocyte, and osteocyte differentiation

MSC differentiation was induced by exchanging StemMACS iPS-Brew XF medium with differentiation medium containing 20% KSR, 1% GlutaMAX, 1% NEAA, and 0.1 mM beta-mercaptoethanol supplemented with 10 μM SB431542 (Miltenyi Biotec). Fresh medium was applied every other day and after 7 days, cells were transferred in a 1:3 ratio to a non-coated tissue culture-treated plate with MSC expansion medium (Miltenyi Biotec). Fresh medium was applied daily before splitting the cells at differentiation day 14. Process control of MSC differentiation was performed by flow cytometry and RT-qPCR at day 21. At day 21, MSCs were differentiated to adipocytes or osteocytes using StemMACS AdipoDiff Media or StemMACS OsteoDiff Media (both Miltenyi Biotec), respectively. Fresh medium was applied every 3 days for 20 days before process control by OilRed O or Alizarin Red staining, respectively.

### Cardiomyocyte differentiation

Cardiomyocytes were generated according to a published protocol [60]. Briefly, 1.0 × 10^6^ cells were dissociated as single cells using Accutase and plated in a well of a 12-well plate with StemMACS iPS-Brew XF and differentiation was induced the following day by changing the medium to RPMI-1640 (Sigma) with 2% B-27 supplement without insulin (Thermo Fisher Scientific) and 10 μM CHIR99021(R&D Systems). Same medium was used the following day, and at day 3, half of the medium was replaced with RPMI/B-27 without insulin supplemented with 10 μM IWP-2 (Santa Cruz Biotechnology). At days 5 and 7, RPMI/B-27 first without insulin and then with full B-27 (Thermo Fisher Scientific) was used. Fresh medium was applied after 3 days and cultures beginning to contract around day 12 were used for experiments. Process control of lateral mesoderm markers was performed at day 3.

### Nephron differentiation

The protocol for differentiation of nephrons was optimized based on a published protocol [61]. Starting with undifferentiated cell cultures of ~ 70% confluency, a medium containing RPMI-1640, 1% GlutaMAX and 2% B-27 supplement (basal medium), 10 μM CHIR99021, and 500 nM dorsomorphin (Tocris) was used. Fresh medium was applied every other day and from day 4 onwards, the basal medium was supplemented with 10 ng/ml of Activin A (R&D Systems). At day 7, basal medium was supplemented with 10 ng/ml FGF9 (R&D Systems) and at day 9, with 3 μM CHIR99021 in addition for 48 h. Afterwards, basal medium supplemented with FGF9 was applied daily until day 21. Process controls were performed at day 7 for intermediate mesoderm markers, at day 14 for nephron progenitor markers, and at day 21 for nephron markers by RT-qPCR and immunostaining.

### Definitive endoderm, lung progenitor, and hepatocyte differentiation

The protocol for differentiation of definitive endoderm was based on a published protocol [62]. Briefly, hPSCs were dissociated using Accutase and 4 × 10^5^ single cells were seeded in a Matrigel-coated 24-well in RPMI-1640 medium, supplemented with 2% B-27, 50 U/ml of penicillin/streptomycin (Pen/Strep; Thermo Fisher Scientific), 100 ng/ml Activin A, 1 μM CHIR99021, and 10 μM Y-27632. Fresh medium was applied daily until day 6 without Y-27632, but with 0.25 mM sodium butyrate (Sigma) on the first day and 0.125 mM afterwards. Process controls were performed at day 6 by flow cytometry and RT-qPCR.

Subsequent differentiation towards lung progenitor cells was adapted from a published protocol [63]. Briefly, foregut endoderm was induced using day 6 definitive endoderm cells by DMEM/F-12 medium, supplemented with 1% GlutaMAX, 2% B-27, 1% N-2 (Thermo Fisher Scientific), 50 U/ml Pen/Strep, 0.05 mg/ml of l-ascorbic acid (Sigma), 0.4 mM of monothioglycerol (Sigma) (basal medium), 2 μM dorsomorphin, and 10 μΜ SB431542. Fresh medium was applied daily and on day 10, lung progenitor differentiation was induced by applying basal medium supplemented with 20 ng/ml recombinant human BMP4 (R&D Systems), 50 nM retinoic acid (Sigma), and 3 μΜ CHIR99021. Fresh medium was applied daily until differentiation day 15 when expression of *NKX2.1* was observed.

Hepatocyte differentiation was based on a published protocol [64]. Briefly, 1.5 × 10^5^ definitive endoderm cells were dissociated with Accutase, transferred to a Matrigel-coated 24-well plate, and treated by DMEM/F-12 with 10% KSR, 1% NEAA, 1% GlutaMAX, and DMSO (Sigma) together with 10 μM Y-27632 and 100 ng/ml recombinant human hepatocyte growth factor (R&D Systems). Medium was changed daily without Y-27632 for 10 days, and process controls were conducted by RT-qPCR and immunofluorescence.

### Neural stem cell differentiation

The protocol for differentiation of neural stem cells (NSCs) was based on the generation of neurospheres [65]. Briefly, hESCs were harvested using a 2 mg/ml collagenase IV solution (Thermo Fisher Scientific) and resuspended in DMEM/F-12 medium supplemented with 20% KSR, 1% NEAA, 1% GlutaMAX, 10 μM SB431542, 5 μM dorsomorphin, 20 μM CHIR99021, 10 μM purmorphamine (Miltenyi Biotec), and 10 μM Y-27632, and plated on an ultra-low attachment 6-well plate (Corning). Fresh medium was applied without Y-27632. 48 hours later, the basal medium was exchanged with N2B27-based medium containing a 1:1 mixture of DMEM-F-12 and Neurobasal A (Thermo Fisher Scientific) with 0.5% N-2, 1% B-27 minus vitamin A, 1% NEAA, and 1% GlutaMAX, and the small molecules described above. At day 5, N2B27-based medium supplemented with 50 μg/ml l-ascorbic acid, SB431542, and dorsomorphin was applied. At day 7, the neurospheres were mechanically dissociated and plated on Matrigel-coated plates. 24 hours before the replating, the medium was supplemented additionally with 5 ng/ml bFGF (Peprotech). Plated neurospheres were maintained for 7 days using the same medium and on day 14, confluent neuroepithelial outgrowths were passaged in a 1:10 dilution using collagenase IV. The NSC cultures were passaged every 7 days and maintained in N2B27 medium with SB431542, dorsomorphin, and bFGF at same concentrations as above with medium change every other day. Process control of NSC differentiation was performed at day 21 which we define as passage 1.

### Astrocyte differentiation

The protocol of astrocyte differentiation was based on a published protocol [66]. Briefly, tissue culture-treated plates were coated for 2 h with 10 ng/ml laminin/poly-l-ornithine (Sigma), and day 21 NSCs were dissociated using Accutase and plated at a ratio of 2.8 × 10^5^ cells per well of a 12-well plate with N2B27 medium supplemented with 20 ng/ml bFGF, 10 ng/ml BMP4, and 5 ng/ml CNTF (R&D Systems). On day 15, medium was supplemented with 10 ng/ml bFGF, 10 ng/ml EGF (Sigma), and 10 ng/ml neuregulin (R&D Systems), and the cells were differentiated for additional 15 days and then analyzed.

### Motor neuron differentiation

The motor neuron differentiation was based on a published protocol [67]. Briefly, plates were coated, first with 10 ng/ml laminin, poly-l-ornithine, collagen I, and collagen IV (Sigma) for 1 h each and then with 10 ng/ml vitronectin (Peprotech) for 1 h. Instead of vitronectin, 10 ng/ml fibronectin (Sigma) was used for later passaging. 1.5 × 10^5^ day 21 NSCs were seeded per well of a 12-well plate with N2B27 medium supplemented with 100 ng/ml SHH, 10 ng/ml BDNF, 10 ng/ml GDNF, 10 ng/ml IGF (all from R&D System), and 100 nM retinoic acid. After 15 days, the medium was supplemented with 0.1 μM y-secretase inhibitor XXI (Merck) and 0.1 μM cAMP (Sigma Aldrich). Cells were analyzed at day 75.

### Cortical neuron differentiation

The protocol of cortical neuron differentiation was based on a previously published protocol [68], with minor modifications. Briefly, iPSCs were plated in a 1:1 mixture of DMEM/F-12 and Neurobasal A, 1% N-2, 2% B-27, 1% GlutaMAX, 1% NEAA, 1000 U/mL Pen/Strep, 5 μg/ml human insulin (Thermo Fisher Scientific), and 0.1 mM β-mercaptoethanol with 10 μM SB431542 and 1 μM dorsomorphin, and fresh media was applied daily. At day 10, cells were dissociated with Accutase and plated on poly-l-ornithine- (1:1000) and laminin- (1:200) coated plates at 1:4 dilution with the same medium supplemented with 10 μM Y-27632. From the next day onwards, the cells were treated by medium without SB431542 and dorsomorphin. Cells were passaged every 6 days. Process control for neural induction and cortical neuron progenitor differentiation was performed after 15 and 35 days.

### Somatic cell lines

Somatic cell lines used in this study were GIBCO® Human Skeletal myoblasts that were cultured for 2 days in DMEM (Thermo Fisher Scientific) together with 2% horse serum (Thermo Fisher Scientific) which induced differentiation to myotubes. Additionally, primary human epidermal keratinocytes (ATCC® PCS200011™), primary adult human dermal fibroblasts (ATCC® PCS201012™), primary human neonatal foreskin fibroblasts (ATCC® CRL-2522™), and primary human astrocytes (ScienCell™ Research Laboratories, #1800) were cultured according to the provider’s instructions.

### Derivation of murine mesenchymal stem cells

Cultures of murine mesenchymal stem cells were established from the femoral bone marrow of female FVB/N mice (Charles River Laboratories, Sulzbach, Germany) by aspiration from the marrow cavity with 1 ml ice-cold PBS and a 0.4-mm injection needle. A solution of single cells was produced by pipetting, filtering through a 70-μm cell strainer (BD), and 5 min centrifugation at 300*g*. Cells were plated in 12 ml of DMEM/F-12 with 1 g/l glucose, 10% MSC-qualified FBS (Thermo Fisher Scientific), 1% GlutaMAX, and 10 μM Y-27632 in T75 cell culture flasks. Cells were kept under hypoxic conditions (2% O_2_, 5% CO_2_) at 37 °C in a humidified atmosphere. Non-adherent cells were depleted by exchanging the medium 2 and 4 h after initial plating, whereas later on, fresh medium was applied every 3.5 days. When reached approximately 80% confluency, cells were passaged in a 1:3 ratio using Accutase.

### Derivation of primary murine astrocytes

Primary mouse astrocytes of the C56BL/6 P3 strain were derived from whole cortex preparations. The brain was washed with HBSS (Sigma) supplemented with 50 U/ml Pen/Strep, and meninges and blood vessels were removed. The cortex was isolated and cut into smaller pieces and further resuspended in 10 ml HBSS/Pen/Strep. The minced tissue was plated on poly-d-lysine-coated plates (40 μg/ml, 1 h incubation) in DMEM/F-12 supplemented with 10% FBS, 50 U/ml Pen/Strep, 10 ng/ml FGF2, and 10 ng/ml EGF. Fresh medium was applied every other day until the culture became confluent.

### Derivation of primary murine cardiomyocytes

Primary mouse cardiomyocytes cultures were prepared using the Primary Cardiomyocyte Isolation Kit (Thermo Fisher Scientific) according to the manufacturer’s instructions.

### Derivation of primary murine hepatocytes

The protocol of primary hepatocyte derivation was based on a published protocol [69]. Liver was obtained from 14-week-old C56BL/6 mice and digested using 2 mg/ml collagenase IV solution (Thermo Fisher Scientific) at 37 °C for 45 min. The digested tissue was plated in a 10-cm dish with Williams E medium (Sigma) supplemented with 5% FBS and mechanically dissociated. Then, cells were filtered using a 70-μm cell strainer, and 6 ml cell suspension was layered on top of a Percoll (Sigma) gradient of 1.12 g/ml, 1.08 g/ml, and 1.06 g/ml in PBS. Cells were centrifuged for 20 min at 800*g* and washed with Williams E medium with 5% FBS. After another centrifugation at 300*g* for 10 min, the cells were resuspended in Williams E medium with 5% FBS, 1% GlutaMAX, 50 U/ml Pen/Strep, 50 ng/ml EGF, 1 μg/ml insulin, 10 μg/ml transferrin (Sigma), and 1.3 μg/ml of hydrocortisone (Sigma) and plated on 10 μg/ml rat tail collagen I- (Sigma) coated plates with daily medium change.

### Oil Red O staining

Following adipocyte differentiation, cells were washed twice with PBS, fixed with 10% neutral buffered formalin (Sigma) for 45 min, then washed twice with tap water and fixed again with 2-propanol (Sigma) for 5 min. Filtered Oil Red O solution (1.8 mg/ml in 2-propanol; Sigma) was added to the cells and incubated for 10 min. After two washes with PBS, cells were counterstained with Mayer’s hematoxylin solution (Sigma) for 3 min, before two washes with tap water, addition of PBS, and imaging with a phase-contrast microscope. All steps were performed at RT.

### Alizarin Red staining

Following osteocyte differentiation, cells were washed twice with PBS and fixed with 10% neutral buffered formalin (Sigma) for 45 min. Next, cells were washed twice with tap water and incubated with filtered Alizarin Red staining solution (20 mg/ml; Sigma) for 45 min. After four washes with deionized water, PBS was added to the cells and images were obtained with a phase-contrast microscope. All steps were performed at RT.

### Immunofluorescence staining

Cells were grown on imaging slides (Ibidi), washed three times with PBS, and fixed with 4% paraformaldehyde (Sigma) in PBS for 10 min, followed by three washes using PBS. After permeabilization using 0.5% Triton-X-100 (Sigma) in PBS at 4 °C overnight and three washes with PBS, slides were blocked with 0.1% Triton-X-100 and 1% FBS in PBS for 1 h at room temperature. Incubation with primary antibodies was performed at 4 °C overnight. After three washes with PBS, slides were incubated with the species-corresponding secondary antibodies (Thermo Fisher Scientific) for 2 h at room temperature in the dark and washed three times with PBS afterwards. The samples were mounted with ProLong® Gold Antifade Reagent with DAPI (Thermo Fisher Scientific) on a coverslip and imaged with an Axio Observer.Z1 inverted epifluorescence microscope (Zeiss) equipped with a × 10/0.3 Plan-NEOFLUAR objective (Zeiss). Primary antibodies were diluted 1:100 unless stated otherwise and secondary antibodies 1:1000 in blocking buffer. Primary antibodies that were used in this study are listed in Additional file 5: Table S2.

### Single molecule fluorescence in situ hybridization 

Cells were plated on imaging slides (Ibidi), fixed with 4% paraformaldehyde, washed twice with PBS, and permeabilized with 70% ethanol overnight at 4 °C. After two washes with PBS and pre-hybridization solution (10% deionized formamide (Merck Millipore), 2x SSC), slides were incubated with 50 μl hybridization solution containing 2x SSC, 10% formamide, 50 μg competitor *E. coli* tRNA (Roche Diagnostics), 10% dextran sulfate (VWR), 2 mg/ml BSA (UltraPure; Life Technologies), 10 mM vanadyl-ribonucleoside complex (NEB) and 1 ng/μl smFISH probes for 6 h at 37 °C. Afterwards, slides were washed twice with pre-hybridization solution at 37 °C, then twice with PBS with subsequent mounting with ProLong® Gold Antifade Reagent with DAPI. Slides were imaged after 12 h when the mounting medium was fully cured on an Axio Observer.Z1 inverted epifluorescence microscope equipped with a × 63/1.4 Plan-APOCHROMAT objective (Zeiss).

Probe Designer software by Biosearch Technologies was used to design probes for *hNEAT1* 5′ segment and *mNEAT1* middle segment, both conjugated to Quasar®670 fluorescent dye. Sequences are listed in Additional file 5: Table S4. Probes for *hNEAT1* middle segment, *mNEAT1* 5′ segment, and *MALAT1* (all conjugated to Quasar®570) were pre-designed by Biosearch Technologies.

### Chemicals used for DNA binding

Cells were treated either by 2 μM actinomycin D (Thermo Fisher Scientific), 100 μg/ml Hoechst 33342 (Thermo Fisher Scientific), 50 μM α-amanitin (Cayman Chemical), and 5 μM mithramycin A (Abcam). Vincristine (Selleckchem), etoposide (Selleckchem), and flavopiridol (Biomol) were used at concentrations specified in Fig. 4f.

### Image analysis for paraspeckle counting

The spot detection program *Airlocalize* [70] was used for paraspeckle quantification based on 3D image stacks with 6-μm depth in 0.3-μm increments as described previously [18]. The average number of paraspeckles was calculated from images containing 10–150 cells. Seven images were analyzed per condition and replicate.

### Quantification of nucleus size

Quantification of nucleus size based on DAPI staining was done using the *Fiji* software. Per image, an intensity threshold was determined to mask the DAPI staining in a maximum projection of a 3D image stack with 6 μm depth. The total DAPI area was divided by the number of cells per image to determine the average nucleus size per cell per image. The determination of nuclear size in single cells (Fig. 3b) was done by manually masking DAPI labeled nuclei and analyzing the nuclear area by the “Analyse Particles” function in *Fiji*.

### Flow cytometry analysis

Surface marker staining was performed by washing dissociated cells with FACS buffer (1% FBS in PBS), centrifugation, removal of supernatant and incubation with primary antibodies in FACS buffer for 30 min on ice. Next, after centrifugation and removal of supernatant, cells were incubated with species-corresponding secondary antibody for 30 min on ice, before washing and final resuspension in FACS buffer. A similar protocol was carried out with primary antibodies that were already conjugated to fluorophores.

Intracellular staining was performed according to instructions of the Inside Stain Kit (Miltenyi Biotec). Primary antibodies were incubated for 1 h at room temperature with 2.0 × 10^5^ cells. Secondary antibodies were incubated for 30 min on ice. Cells were washed once with Inside Perm solution before resuspending them in FACS buffer for analysis.

Unconjugated primary antibodies were diluted 1:100 and secondary antibodies 1:1000 in FACS buffer. Samples were analyzed using the BD FACSAria III cell sorter (BD Biosciences), and data was processed using FlowJo software.

### RNA extraction and quantitative RT-PCR (RT-qPCR)

RNA extraction was performed using the RNeasy Mini Kit (Qiagen) according to the manufacturer’s instructions. Reverse transcription was performed using the Verso cDNA Synthesis Kit (Thermo Fisher Scientific) with 200 ng RNA per reaction. RT-qPCR was performed in 384-well plates using 5 μl of SYBR Green PCR Master Mix (Thermo Fisher Scientific), 1 μl cDNA, and 1 μl of 5 μM primer forward and reverse mix in a 10 μl reaction. PCR conditions were 2 min at 50 °C and 10 min at 95 °C followed by 40 cycles of 15 s at 95 °C and 1 min at 60 °C. Relative expression levels were calculated using the Delta-Delta Ct method normalized with *GAPDH*. Statistical analysis was performed with the GraphPad Prism 7 software. RT-qPCR primers are listed in Additional file 5: Table S3.

### Generation of *NEAT1*^*−/−*^, *NEAT1ΔTH,* and *NEAT1*^*STOP*^ hESCs

Generation of *NEAT1*^*−/−*^ and *NEAT1ΔTH* clones from hESCs was carried out according to a published protocol [71]. Briefly, the Protospacer adjacent Motif (PAM) sequence was identified using the crispr.mit.edu website. *BbsI*-digested pSpCas9(BB)-2A-GFP vector (Addgene plasmid ID: 48138) was ligated with annealed forward/reverse guide RNA (gRNA) mix (1:250 dilution) using T4 ligase (NEB). NEB® 5-alpha competent *E. coli* bacteria (NEB) were inoculated with ligated plasmid and plated on agar plates. Bacteria colonies were propagated and plasmids were isolated using the GeneJET Plasmid MiniPrep Kit (LifeTechnologies) according to the manufacturer’s instructions. Sanger sequencing was used to screen correct integrations. 1.0 × 10^6^ hESCs were nucleofected with 5 μg of up- and downstream gRNA/Cas9 plasmid mix using the P3 Primary Cell 4D-Nucleofector® Kit (Lonza) according to the manufacturer’s instructions. Cells were plated 2 days later, and single clones were picked and analyzed for successful genomic deletion by PCR. *NEAT1*^*STOP*^ hESCs were generated by inserting a polyA stop cassette approximately 1500 base pairs after the *NEAT1* transcription start site. 1.0 × 10^6^ hESCs were transfected with 4 μg donor, and 2 μg gRNA plasmid and clones were tested for successful integration by PCR. Guide RNAs and primers for PCR-based screening are listed in Additional file 5: Table S1.

### DNA extraction and polymerase chain reaction (PCR)

Isolation of genomic DNA for screening of KO clones after transfection of CRISPR/Cas9 was performed using 30 μl QuickExtract™ (Biozym) according to the manufacturer’s instructions. PCR was performed using Q5 Polymerase mastermix (NEB) with 100 ng DNA.

## Supplementary information

Additional file 1: **Figure S1.** Related to Figs. 1 and 2, characterization of germ layer progenitors and differentiated cells. **a** Analysis of mesoderm differentiation towards mesenchymal stem cells (MSCs) showing the expression of characteristic markers CD73 and CD90 [72]. **b-d** Differentiation towards definitive endoderm showing the upregulation of CXCR4, EPCAM and CD117 cell surface markers (**b**) and a cohort of characteristic markers as well as the downregulation of pluripotency genes by RT-qPCR (**c**), and the expression of *eGFP* integrated in *NKX2.1* which marks the formation of human lung progenitors [57] (**d**). Scale bar: 10 μm. **e, f** Representative immunocytochemistry images of NSCs showing the expression of characteristic markers PAX6, SOX1 and NESTIN on day 21 of NSC differentiation (**e**), and the cortical neuron progenitor markers FOXG1 and PAX6 (**f**) [68]. Scale bar: 50 μm. **g, h** Oil Red O (**g**) and Alizarin Red (**h**) staining of human MSCs differentiated to adipocytes and osteocytes, respectively. Scale bar: 500 μm. **i, j** Time course RT-qPCR analysis of representative pluripotency, mesoderm and cardiac markers during lateral mesoderm differentiation to cardiomyocytes (**i**) [60], and of representative intermediate mesoderm and nephron progenitor markers during nephron differentiation (**j**) [61]. **k** Representative images showing the expression of characteristic nephron progenitor markers CDH5 and WT1 at day 14 of differentiation. Scale bar: 50 μm. **l** RT-qPCR analysis of representative pluripotency, definitive endoderm and hepatocyte markers during differentiation to hepatocytes at day 16 [64]. **m-o** RT-qPCR analysis of representative pluripotency, motor neuron, glial and cortical markers following differentiation to motor neurons (**m**), astrocytes (**n**) and cortical neurons (**o**). *n* = 2 independent experiments (*n* = 3 in **i, l**), error bars represent standard deviation, cells in different passages were used for replicates.

Additional file 2: **Figure S2.** Related to Fig. 2, quantification of paraspeckles. **a, b** Representative images of *NEAT1_2* (red) in cells representing tissue progenitors (**a**), and terminally differentiated cells (**b**). **c** The number of paraspeckles per cell in progenitors and differentiated cell types used to calculate the average number of paraspeckles in Fig. 2b. Each dot represents the average of one microscopic image displaying 10–150 cells. *n* = 3 independent replicates using cells of different passages were analyzed with 5–7 images per replicate. Changes in number of paraspeckles are statistically significant for all cell types compared to human ESCs (*p* < 0.0001, unpaired t-test; *** *p* < 0.001). **d** Number of paraspeckles in neural stem cells (NSCs) in passage (P) 1, 3 and 5 representing respectively differentiation day 21, 35 and 49. Counting as in **c**. *n* = 2 independent differentiation experiments. n.s. = not significant. **e** Correlation of differentiation time as specified in the method section and averaged number of paraspeckles per cell type. **f** Representative images of *NEAT1_2* (red) in mouse ESCs and primary cardiomyocytes, hepatocytes, MSCs and astrocytes, next to same cell types from the human. **g** Correlation of *NEAT1_2* total intensity and the number of paraspeckles per cell in representative human and mouse cell types. Each point represents a microscopic image. h RT-qPCR of *NEAT1_2* in 19 cell types and correlation with averaged number of paraspeckles per cell indicated in Fig. 2b. RNA was obtained from 2 - 4 independent RNA differentiation experiments of cells in different passages. **i** Time-course RT-qPCR analysis of endogenous transcription of pluripotency factors OCT4, SOX2 and NANOG during reprogramming of human neonatal fibroblasts. *n* = 2 independent reprogramming experiments. **j,k** Representative brightfield (**j**) and *NEAT1_2* (**k**) images taken during fibroblast reprogramming. *n* = 2 independent reprogramming experiments using cells of different passages were analyzed with 7 images per replicate; nascent iPSC colonies are marked with white circles. Error bars represent standard deviation. DAPI staining in blue; scale bar is 10 μm in smFISH images and 50 μm in brightfield images. r in **e,g**, **h** represents the Pearson’s correlation coefficient.

Additional file 3: **Figure S3.** Related to Fig. 4, characterization of lncRNA foci after treatment by Actinomycin D. **a** Representative images of *NEAT1_2* smFISH after treatment of human ESC derived astrocytes, definitive endoderm cells, NSCs and primary neonatal fibroblasts by 2 μM ActD. **b** Immunocytochemistry of nucleolar protein fibrillarin (FBL) and paraspeckle proteins SFPQ and NONO in untreated trophoblast progenitors and after treatment by 2 μM ActD for 1 h. **c** Representative immunocytochemistry images of γ-H2AX foci indicating DNA double-strand breaks in trophoblast progenitors and after addition of small DNA binding molecules. Quantification in Fig. 4e. Concentrations as in Fig. 4a, b. **d** A table indicating the potential of small molecules used in this study to bind DNA, to inhibit transcription and to disintegrate paraspeckles. **e, f** Representative images (**e**) and quantification (**f**) of *MALAT1* smFISH in human trophoblast progenitors treated with ActD as above. *n* = 2 independent replicates with 7 images per replicate. Dashed lines in **a** and **f** show the locations of the borders of the nuclei. Scale bar is 10 μm.

Additional file 4: **Figure S4.** Related to Fig. 5, characterization of *NEAT1*-manipulated cells. **a** RT-qPCR of pluripotency and differentiation markers of undifferentiated *NEAT1*^*−/−*^, *NEAT1*^*STOP*^ and *NEAT1ΔTH* hESC clones. **b, c** Flow cytometry analysis of pluripotency surface markers TRA1–60 and SSEA5 after 2 days of spontaneous differentiation of WT, *NEAT1ΔTH* and *NEAT1*^*−/−*^ hESCs. **d** RT-qPCR time course analysis of pluripotency and neural marker genes during differentiation towards neural rosettes which appeared around day 12 of the differentiation towards NSCs. Same cell lines as in **b, c**. **e-g** RT-qPCR analysis of *NEAT1ΔpA* hESC clones differentiated to lateral mesoderm (**e**), definitive endoderm (**f**) and neuroectoderm by 4 days differentiation of NSCs (**g**). **h-k** Representative histograms and quantification of flow cytometry analysis for pluripotency markers in pluripotent (**h, j**) *NEAT1*^*STOP*^ hESCs and after 3 days of spontaneous differentiation (**i, k**). Forward and side scatter gating was employed to gate out debris and cell clumps. n (# of experiments / # of clones) = 3/2 in **a**, 1/3 in **c, e, f**, 2/3 in **d,** **g** and 2/2 in **j, k**. Error bars represent standard deviation.

Additional file 5: List of primers, smFISH probes and antibodies. Table S1. Sequence and genomic location of gRNAs and primers used for the generation of CRISPR lines. Table S2. List of antibodies. Table S3. List of RT-qPCR primer sequences. Table S4. List of sequences of smFISH probes.

Additional file 6: Raw data for graphs with *N* < 6. Table S5. Raw data for Fig. 1b-d, f. Figure S1c, i, j, l-o, Figure S2h, i. Table S6. Raw data for Fig. 5d, e, Figure S4a, d-g. Table S7. Raw data for Fig. 5g, i, Figure S4c, j, k.

## Data Availability

The datasets supporting the conclusions of this article are included within the article (and its additional files). Raw data for experiments with *N* < 6 can be found in Additional file 6: supplementary Tables 5–7.
