# Supplementary figures and images for: Nucleus size and DNA accessibility are linked to the regulation of paraspeckle formation in cellular differentiation
Source: BMC Biol. 2020 Apr 22;18:42. doi: 10.1186/s12915-020-00770-y (PMC7178590; doi:10.1186/s12915-020-00770-y)

**Figure S1**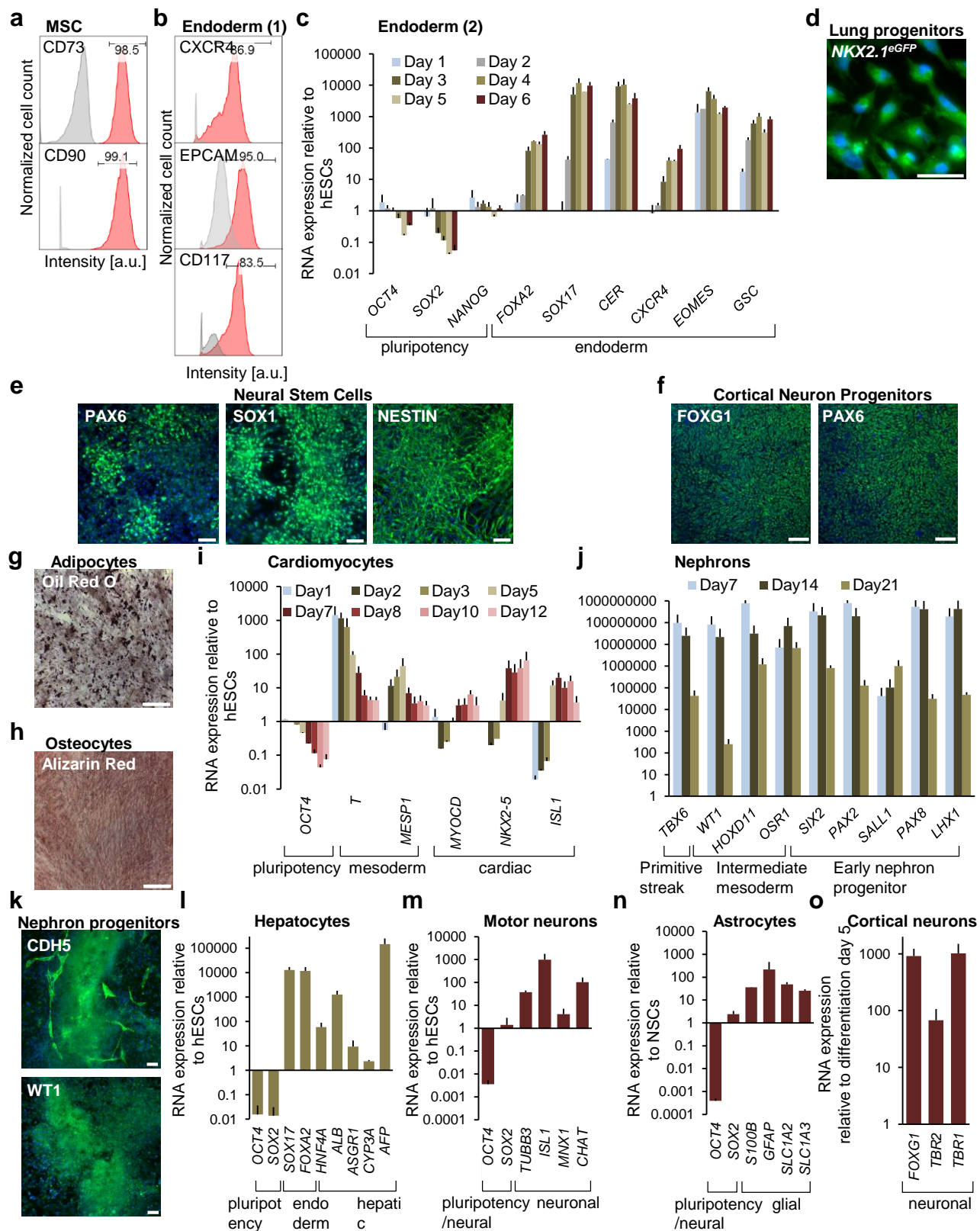

Supplement: Supplementary file 1 — Figure S1. Related to Figs. 1 and 2, characterization of germ layer progenitors and differentiated cells. a Analysis of mesoderm differentiation towards mesenchymal stem cells (MSCs) showing the expression of characteristic markers CD73 and CD90 [72]. b-d Differentiation towards definitive endoderm showing the upregulation of CXCR4, EPCAM and CD117 cell surface markers (b) and a cohort of characteristic markers as well as the downregulation of pluripotency genes by RT-qPCR (c), and the expression of eGFP integrated in NKX2.1 which marks the formation of human lung progenitors [57] (d). Scale bar: 10 μm. e, f Representative immunocytochemistry images of NSCs showing the expression of characteristic markers PAX6, SOX1 and NESTIN on day 21 of NSC differentiation (e), and the cortical neuron progenitor markers FOXG1 and PAX6 (f) [68]. Scale bar: 50 μm. g, h Oil Red O (g) and Alizarin Red (h) staining of human MSCs differentiated to adipocytes and osteocytes, respectively. Scale bar: 500 μm. i, j Time course RT-qPCR analysis of representative pluripotency, mesoderm and cardiac markers during lateral mesoderm differentiation to cardiomyocytes (i) [60], and of representative intermediate mesoderm and nephron progenitor markers during nephron differentiation (j) [61]. k Representative images showing the expression of characteristic nephron progenitor markers CDH5 and WT1 at day 14 of differentiation. Scale bar: 50 μm. l RT-qPCR analysis of representative pluripotency, definitive endoderm and hepatocyte markers during differentiation to hepatocytes at day 16 [64]. m-o RT-qPCR analysis of representative pluripotency, motor neuron, glial and cortical markers following differentiation to motor neurons (m), astrocytes (n) and cortical neurons (o). n = 2 independent experiments (n = 3 in i, l), error bars represent standard deviation, cells in different passages were used for replicates. [file 12915_2020_770_MOESM1_ESM.tif]

**Figure S2**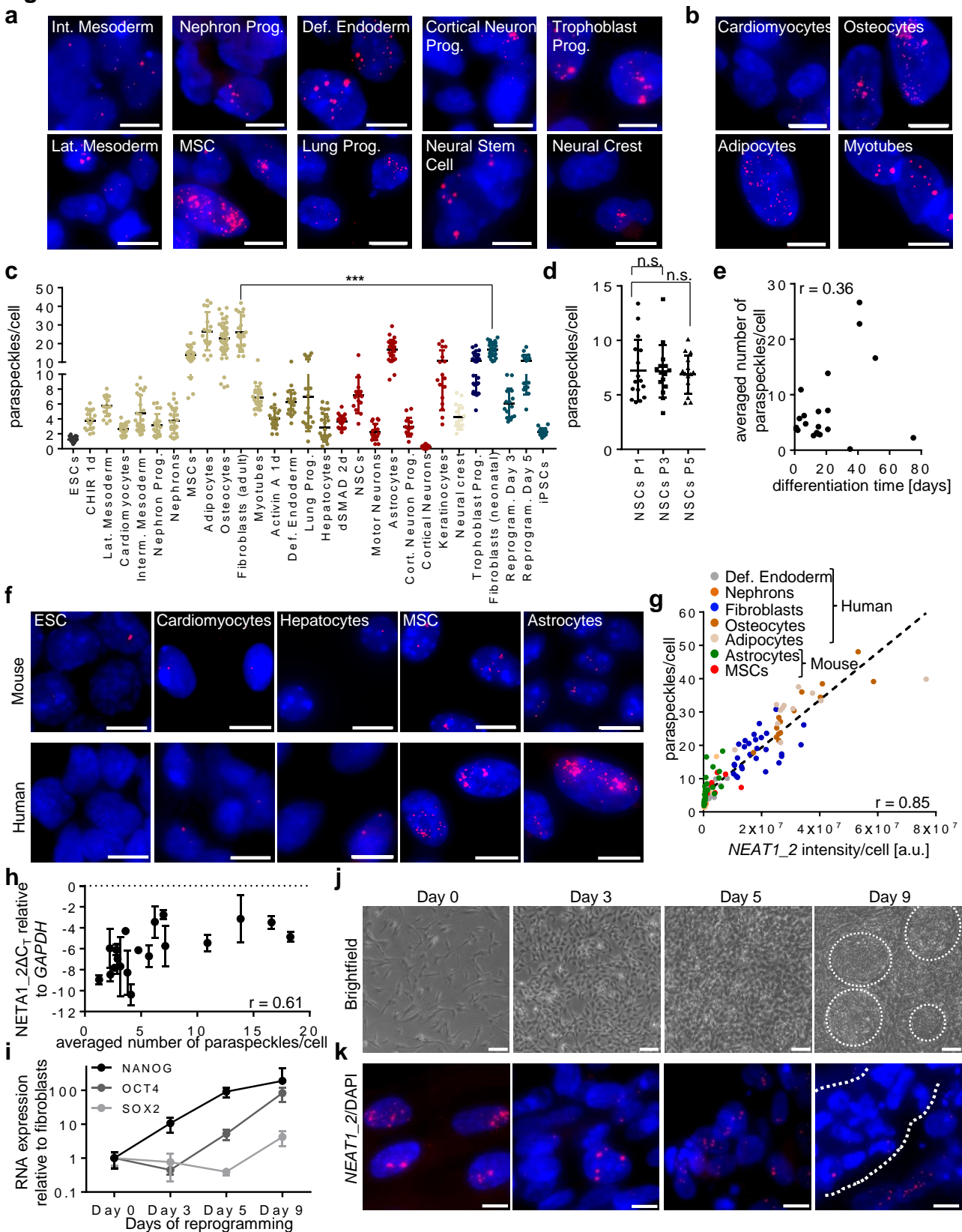

Supplement: Supplementary file 2 — Figure S2. Related to Fig. 2, quantification of paraspeckles. a, b Representative images of NEAT1_2 (red) in cells representing tissue progenitors (a), and terminally differentiated cells (b). c The number of paraspeckles per cell in progenitors and differentiated cell types used to calculate the average number of paraspeckles in Fig. 2b. Each dot represents the average of one microscopic image displaying 10–150 cells. n = 3 independent replicates using cells of different passages were analyzed with 5–7 images per replicate. Changes in number of paraspeckles are statistically significant for all cell types compared to human ESCs (p < 0.0001, unpaired t-test; *** p < 0.001). d Number of paraspeckles in neural stem cells (NSCs) in passage (P) 1, 3 and 5 representing respectively differentiation day 21, 35 and 49. Counting as in c. n = 2 independent differentiation experiments. n.s. = not significant. e Correlation of differentiation time as specified in the method section and averaged number of paraspeckles per cell type. f Representative images of NEAT1_2 (red) in mouse ESCs and primary cardiomyocytes, hepatocytes, MSCs and astrocytes, next to same cell types from the human. g Correlation of NEAT1_2 total intensity and the number of paraspeckles per cell in representative human and mouse cell types. Each point represents a microscopic image. h RT-qPCR of NEAT1_2 in 19 cell types and correlation with averaged number of paraspeckles per cell indicated in Fig. 2b. RNA was obtained from 2 - 4 independent RNA differentiation experiments of cells in different passages. i Time-course RT-qPCR analysis of endogenous transcription of pluripotency factors OCT4, SOX2 and NANOG during reprogramming of human neonatal fibroblasts. n = 2 independent reprogramming experiments. j,k Representative brightfield (j) and NEAT1_2 (k) images taken during fibroblast reprogramming. n = 2 independent reprogramming experiments using cells of different passages were analyzed with 7 images per rep [file 12915_2020_770_MOESM2_ESM.tif]

**Figure S3**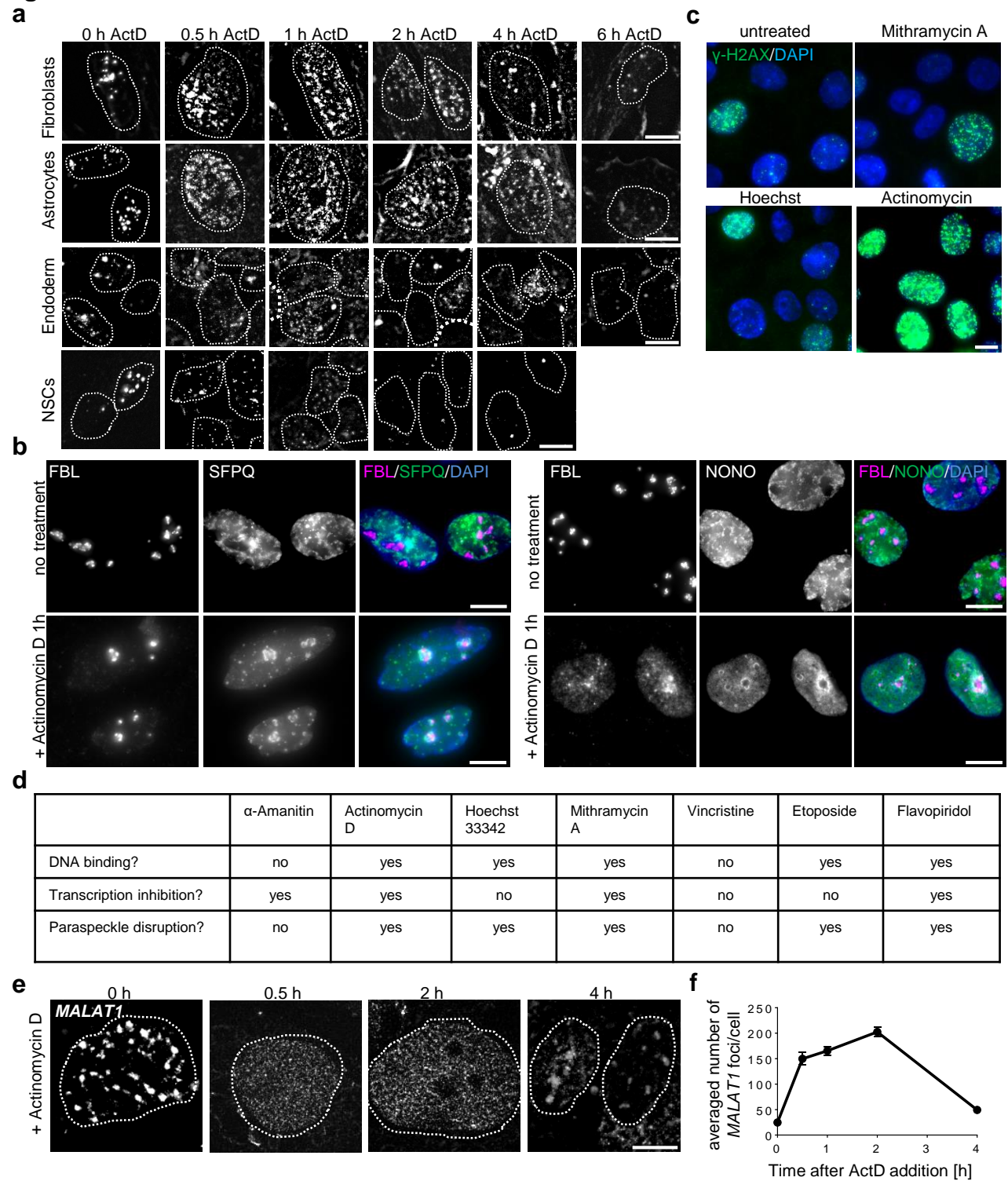

Supplement: Supplementary file 3 — Figure S3. Related to Fig. 4, characterization of lncRNA foci after treatment by Actinomycin D. a Representative images of NEAT1_2 smFISH after treatment of human ESC derived astrocytes, definitive endoderm cells, NSCs and primary neonatal fibroblasts by 2 μM ActD. b Immunocytochemistry of nucleolar protein fibrillarin (FBL) and paraspeckle proteins SFPQ and NONO in untreated trophoblast progenitors and after treatment by 2 μM ActD for 1 h. c Representative immunocytochemistry images of γ-H2AX foci indicating DNA double-strand breaks in trophoblast progenitors and after addition of small DNA binding molecules. Quantification in Fig. 4e. Concentrations as in Fig. 4a, b. d A table indicating the potential of small molecules used in this study to bind DNA, to inhibit transcription and to disintegrate paraspeckles. e, f Representative images (e) and quantification (f) of MALAT1 smFISH in human trophoblast progenitors treated with ActD as above. n = 2 independent replicates with 7 images per replicate. Dashed lines in a and f show the locations of the borders of the nuclei. Scale bar is 10 μm. [file 12915_2020_770_MOESM3_ESM.tif]

**Figure S4**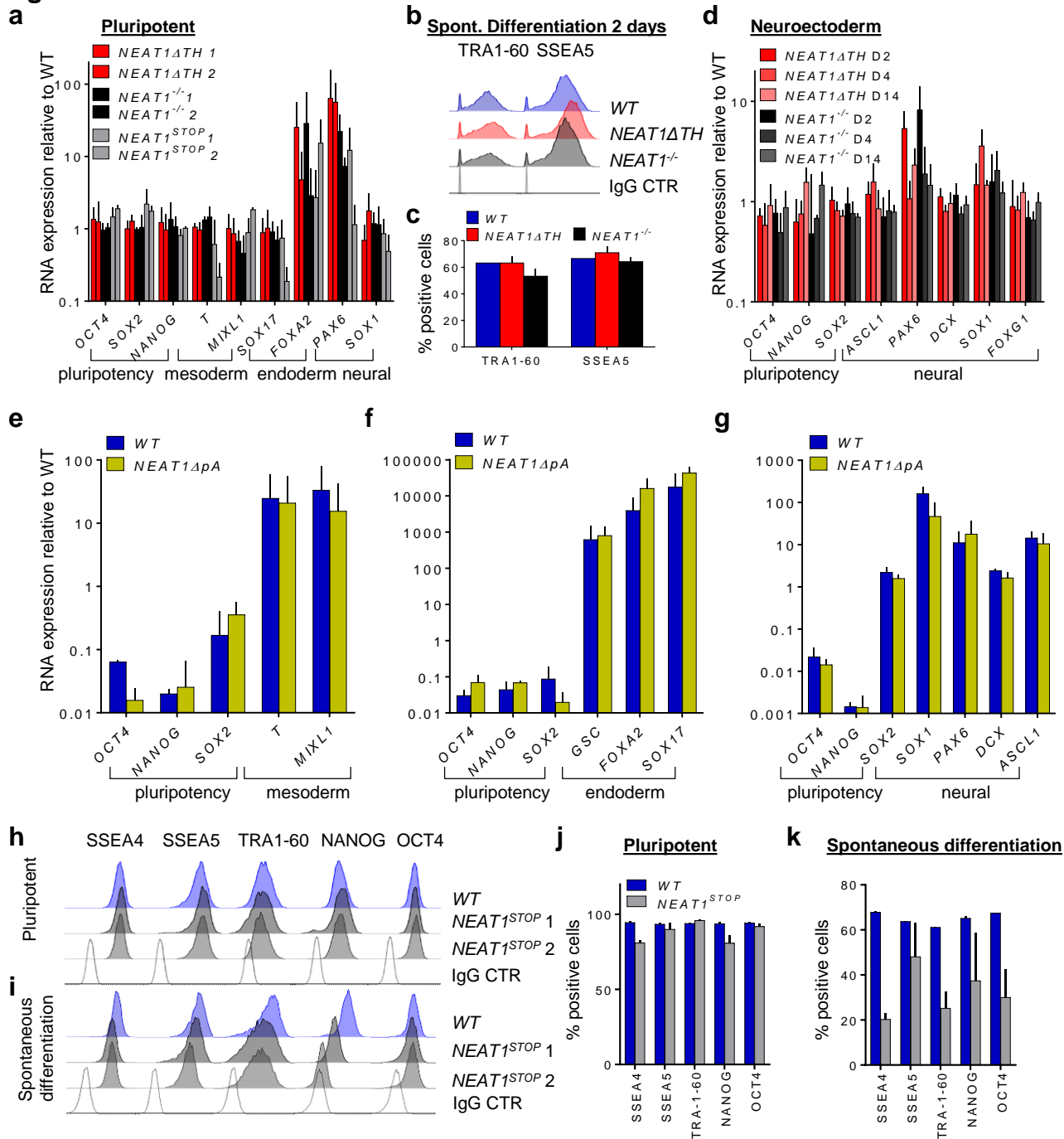

Supplement: Supplementary file 4 — Figure S4. Related to Fig. 5, characterization of NEAT1-manipulated cells. a RT-qPCR of pluripotency and differentiation markers of undifferentiated NEAT1−/−, NEAT1STOP and NEAT1ΔTH hESC clones. b, c Flow cytometry analysis of pluripotency surface markers TRA1–60 and SSEA5 after 2 days of spontaneous differentiation of WT, NEAT1ΔTH and NEAT1−/− hESCs. d RT-qPCR time course analysis of pluripotency and neural marker genes during differentiation towards neural rosettes which appeared around day 12 of the differentiation towards NSCs. Same cell lines as in b, c. e-g RT-qPCR analysis of NEAT1ΔpA hESC clones differentiated to lateral mesoderm (e), definitive endoderm (f) and neuroectoderm by 4 days differentiation of NSCs (g). h-k Representative histograms and quantification of flow cytometry analysis for pluripotency markers in pluripotent (h, j) NEAT1STOP hESCs and after 3 days of spontaneous differentiation (i, k). Forward and side scatter gating was employed to gate out debris and cell clumps. n (# of experiments / # of clones) = 3/2 in a, 1/3 in c, e, f, 2/3 in d, g and 2/2 in j, k. Error bars represent standard deviation. [file 12915_2020_770_MOESM4_ESM.tif]
